# CULTURAL INFLUENCES ON AMERICAN INDIAN AND ALASKA NATIVE DEMENTIA CAREGIVERS

**DOI:** 10.1093/geroni/igad104.0811

**Published:** 2023-12-21

**Authors:** Steffi Kim, Lexie Jackson

**Affiliations:** University of Alaska Anchorage, Anchorage, Alaska, United States; Pacific University, Forest Grove, Oregon, United States

## Abstract

Associated with aging, Alzheimer’s disease and related dementias (ADRD) are likely having a disproportionate effect on North American Indigenous communities due to longer life expectancies and high rates of chronic diseases. Despite missing prevalence data on ADRD in for Native Elders, it is known that one in five American Indians and Alaska Natives experience subjective cognitive decline and one in four adults are currently caregivers. Similarly, Native Hawaiians and Pacific Islanders (NHPI) are twice as likely to have dementia compared to non-Hispanic Whites. Yet, little is known about the journey of dementia caregivers within American and Alaska Native, Native Hawaiians and Pacific Islanders (NHPI) and how their unique cultural influences shape their caregiving experiences within their communities. We will present the voices from the transcripts of three 3 different qualitative data sets that include 93 American Indians and Alaska Natives, and six Native Hawaiian/Pacific Islander caregivers and elders about their personal experiences with taking care of family or community members with dementia and approaches to care. in their communities. The presentation focuses on the influences on cultural values on the caregiving experience, caregiver access to resources, and generative moments throughout caregiving. Results identify similarities among these groups but also distinct variations, which emphasize the importance of linguistic and culturally tailored research, interventions, and policies. Based on our findings, we outline culturally specific recommendations to improve the caregiving experience for each of these Indigenous groups.

